# Channel and Feature Selection for a Motor Imagery-Based BCI System Using Multilevel Particle Swarm Optimization

**DOI:** 10.1155/2020/8890477

**Published:** 2020-08-01

**Authors:** Yingji Qi, Feng Ding, Fangzhou Xu, Jimin Yang

**Affiliations:** ^1^School of Physics and Electronics, Shandong Normal University, Jinan 250358, China; ^2^Department of Neurosurgery, Shandong Provincial Hospital Affiliated to Shandong First Medical University, Jinan 250021, China; ^3^School of Electronic and Information Engineering (Department of Physics), Qilu University of Technology (Shandong Academy of Sciences), Jinan 250353, China

## Abstract

Brain-computer interface (BCI) is a communication and control system linking the human brain and computers or other electronic devices. However, irrelevant channels and misleading features unrelated to tasks limit classification performance. To address these problems, we propose an efficient signal processing framework based on particle swarm optimization (PSO) for channel and feature selection, channel selection, and feature selection. Modified Stockwell transforms were used for a feature extraction, and multilevel hybrid PSO-Bayesian linear discriminant analysis was applied to optimization and classification. The BCI Competition III dataset I was used here to confirm the superiority of the proposed scheme. Compared to a method without optimization (89% accuracy), the best classification accuracy of the PSO-based scheme was 99% when less than 10.5% of the original features were used, the test time was reduced by more than 90%, and it achieved Kappa values and *F*-score of 0.98 and 98.99%, respectively, and better signal-to-noise ratio, thereby outperforming existing algorithms. The results show that the channel and feature selection scheme can accelerate the speed of convergence to the global optimum and reduce the training time. As the proposed framework can significantly improve classification performance, effectively reduce the number of features, and greatly shorten the test time, it can serve as a reference for related real-time BCI application system research.

## 1. Introduction

The brain-computer interface (BCI) is a communication and control system established between the human brain and a computer or external device without the involvement of the nervous system or muscles. Common brain activity patterns used in BCIs include P300 potentials, steady-state visual evoked potentials (SSVEP), and motor imagery (MI) [1]. Among these, the motor imagery-based BCI system (MI-BCI) involves imagining a moving body part without any actual body movement; this provides a new approach for patients with motor disabilities for effective communication. Similar methods have been widely used for rehabilitation applications [2].

Pattern recognition is an important aspect of the MI-BCI system. However, brain signals contain a large amount of physiological and pathological information, and registered electroencephalogram (EEG) signals are mixed with other brain activity signals, which can overlap in both time and space [3]. As a result, the extracted features contain a lot of redundant and misleading information, thus limiting the accuracy of classification. Furthermore, for enhancing the spatial resolution of EEG recording devices and tracing techniques, the number of signal acquisition channels was increased to 16 leads, 32 leads, and 64 leads or higher [4]. An increase in the number of channels not only increases the spatial resolution but also increases the number of features, thus increasing the running time of classification. Hence, task-related features require proper selection mechanisms. Although a genetic algorithm is used most commonly for task recognition [5, 6], different MI optimization methods have been suggested, such as differential evolution [7], particle swarm optimization [8], concave-convex procedure [9], principal component analysis [10], and correlation-based channel and time window selection [11, 12]. It should be noted that the PSO algorithm is another promising technique with simple computation and rapid convergence characteristics, which has been successfully applied to mechanical engineering optimization, business optimization, and clustering problems [13].

Analysis of the existing motor imagery recognition schemes revealed several drawbacks. Firstly, high variance among the optimized feature components may result in low classification performance in some schemes, due to the inability to identify and filter out all misleading features. Secondly, the number of optimized features is still large, which requires a lot of testing time and limits practical application of these schemes. Finally, the features and channels have been analyzed separately, not taking into account the fact that the selection of the optimal features depends on the channel used. Even if the best feature set can be identified, more training time is required.

To solve these technical problems, on the one hand, this study introduces an efficient optimization framework based on multilevel PSO (MLPSO). PSO algorithm is used to perform global search in the whole search space in this scheme, and local search is performed by running this algorithm continuously. This allows improving the ability of the procedure to switch from local to global optima. The scheme reduces the number of features and enhances the classification accuracy via selection of the best feature subsets that match the expected potential cortical activity patterns during the MI task, bringing it the advantages of a great range of application and easy implementation. The reason for using MLPSO in feature optimization is that when a particle's current position coincides with the global best position and the particle velocity is not zero, all particles will move to the position rapidly, leading to a rapid convergence of PSO algorithms. However, the algorithm convergence to the local optimal value is not guaranteed. The procedure means that all particles move to the best position found at present; this phenomenon is known as stagnation [14]. However, this problem can be solved by running the optimizer several times for the same cost function. Hence, MLPSO was used to optimize the task related to motor imagery in this study.

On the other hand, for the last problem, according to the proposed signal processing framework, three optimization schemes based on channel and feature selection, channel selection, and feature selection were designed. Among them, channel and feature selection eliminated irrelevant channels through channel selection and then selected features matching the task through feature selection. These steps accelerated the screening of irrelevant features.

**Figure 1:**
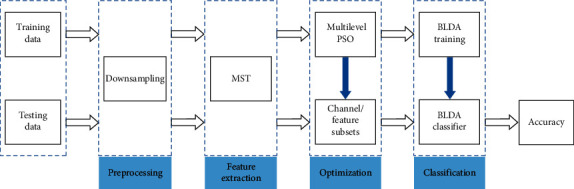
Block diagram of the proposed classification framework.

The current study investigates a signal recognition MI-BCI framework. An MLPSO algorithm was used for optimization in combination with Bayesian linear discriminant analysis (BLDA) classification, and the modified Stockwell transforms (MST) were applied during feature extraction. Three optimization schemes of channel and feature selection, channel selection, and feature selection based on MLPSO optimization are designed for this framework.  Figure 1 shows the block diagram of the proposed methodology framework. Signal processing was implemented with MATLAB, and the simulation was run on a workstation with LINUX Sever, 64 GB of memory, 512 GB of SSD, NVIDIA GeForce TITANX, and six-core Intel(R) Xeon(R) Silver 4114 CPU @ 2.20 GHz.

The remainder of this paper is organized as follows.  Section 2 describes the experimental dataset and preprocessing, feature extraction, classification, multilevel PSO-based channel and feature selection, and classification performance. Sections 3 and 4 present and discuss the classification results of the proposed optimization scheme. Finally, conclusions are summarized at the end of this paper.

## 2. Materials and Methods

### 2.1. Experimental Dataset and Preprocessing

A high-quality signal is an important prerequisite for improving the classification accuracy and evaluating an algorithm's performance. Since an electrocorticogram (ECoG) is recorded on the surface of the cortex and provides higher temporal and spatial resolution, better signal-to-noise ratio, and broader bandwidth compared to those of EEG signals, the BCI Competition III dataset I [15] was used in this study. During the BCI experiment, a subject had to perform imagined movements of either the left small finger or the tongue. The time series of the electrical brain activity was picked up during these trials by using an 8 × 8 ECoG platinum electrode grid, which was placed on the contralateral (right) motor cortex. All recordings were performed using a sampling rate of 1000 Hz. Every trial consisted of either an imagined tongue or an imagined finger movement and was recorded for a duration of 3 seconds.

The dataset consists of 278 trials of training data and 100 trials of test data, which are stored in a 3D matrix named *X* using the following format: trials × electrode channels × samples of time series. The label of the dataset is stored as a vector of -1/1 values named Y. To reduce the amount of data needed for signal processing, the data is downsampled to 100 Hz without causing distortion.

### 2.2. Feature Extraction

Efficient feature extraction method can isolate event characteristics from registered brain signals, thus improving classification performance. The Stockwell transform (ST) is an extension of wavelet transform, based on a moving and scalable localizing Gaussian window, providing frequency-dependent resolution while maintaining a direct connection to the Fourier spectrum [16].

The ST of the time series *x*(*t*) can be obtained as follows:

(1) $$\begin{array}{c} S\left(\tau ,\;f\right)=\overset{+\infty }{\underset{-\infty }{\int }}x\left(t\right)g\left(\tau -t,\;f\right)e^{-i2\pi ft}\text{d}t. \end{array}$$

The Gaussian window *g*(*τ* − *t*,  *f*) is defined by

(2) $$\begin{array}{c} g\left(\tau -t,\;f\right)=\frac{1}{\sqrt{2\pi }\sigma \left(f\right)}e^{-{\left(\tau -t\right)}^{2}/2\sigma^{2}\left(f\right)}, \end{array}$$

and the standard deviation *σ*(*f*) is the function of the frequency *f*, which is equal to

(3) $$\begin{array}{c} \sigma \left(f\right)=\frac{1}{\left|f\right|}. \end{array}$$

By adjusting the time-frequency resolution of the standard deviation of the Gaussian window, MST can provide better energy concentration than ST, obtaining higher-frequency resolution at lower frequencies and better time localization at higher frequency [17]. Accordingly, it has been used to detect dynamic brain signals. The standard deviation of MST is represented as

(4) $$\begin{array}{c} \sigma^{\prime }\left(f\right)=\frac{p}{{\left|f\right|}^{q}}, \end{array}$$

where the scaling factors *p* and *q* determine the width and height of the Gaussian window, respectively.

**Figure 2:**
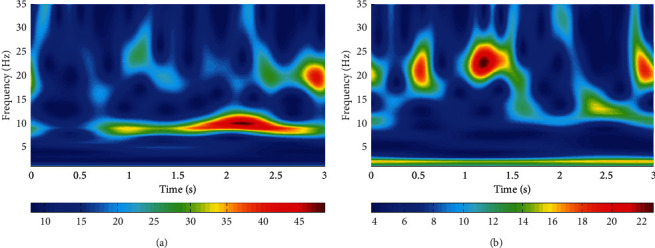
(a, b) MST-based power spectrum features of channel 12 of the first and second trials of the training set, respectively. The 12th channel was chosen because, as described in the channel selection section of this article, the 12th channel was one of the best channel combinations.

According to the frequency range of the MI, the frequency range of the MST is set to 1–35 Hz and the interval is set to 1 Hz. The power spectral density (PSD) is then calculated. Therefore, 35 features were extracted for each channel. Since there were 64 channels, 2240 features were extracted for each trial. Therefore, the number of features of the training set and test set are 278×2240 and 100×2240, respectively. The power spectrum after feature extraction in two trials with different labels in the training set is shown in Figure 2. Observably, the frequency distribution of energy in the two figures is visibly different.

### 2.3. Classification

As an extension of Fisher's linear discriminant analysis, BLDA applies regularization in the training process; it has the advantages of automatically adjusting parameters and avoiding data overfitting in classification [18]. These characteristics make it suitable for real-time BCI systems.

In the dataset, the labels of the samples are denoted by “1” and “−1,” but the output of the classifier is usually not two values. Therefore, the predicted output is changed by setting the threshold to 0; that is, the predicted outputs of experiments that are ≥0 are marked as “1,” and those that are <0 are marked as “−1.”

### 2.4. Multilevel PSO for Channel and Feature Selection

PSO is a population-based optimization algorithm based on the social behavior of bird flocking [19]. The algorithm firstly initializes a group of particles randomly in the given solution space, updating the velocity and position of the particles in the solution space by tracking two “best values.” One “best value” is the best position found by a single particle in iteration, called the personal best position (*pbest*). The other is the global best position (*gbest*) found by all of the particles in the iteration. For the PSO, the particles are calculated based on the following equation:

(5) $$\begin{array}{c} v_{ij}^{k+1}=w*v_{ij}^{k}+c_{1}r_{1}\left(p{\text{best}}_{i}^{k}-x_{ij}^{k}\right)+c_{2}r_{2}\left(g{\text{best}}^{k}-x_{ij}^{k}\right), \end{array}$$

(6) $$\begin{array}{c} x_{ij}^{k+1}=x_{ij}^{k}+v_{ij}^{k+1}, \end{array}$$

where *x*_*ij*_^*k*^ and *v*_*ij*_^*k*^ represent the position and velocity of the *i*-th particle in the *j*-th dimension, respectively, at iteration *k*. *r*_1_ and *r*_2_ are random values between 0 and 1. *c*_1_ and *c*_2_ are the acceleration coefficients. To prevent a blind search of particles and the expansion of the population, the position and velocity are limited to a certain interval [*x*_min_, *x*_max_], [*v*_min_,  *v*_max_]. When values exceeded this range, a boundary absorption strategy was adopted to set the parameters to the adjacent boundary values. *w* is the inertia weight. In this paper, in order to better balance the search ability of the algorithm, the linearly decreasing inertia weight is used; that is,

(7) $$\begin{array}{c} w=w_{\text{max}}-\frac{\left(w_{\text{max}}-w_{\text{min}}\right)*t}{T}, \end{array}$$

where *w*_max_ and *w*_min_ represent the maximum and minimum values of inertia weights, respectively, *t* is the current iteration, and *T* represents the maximum number of iterations.

**Figure 3:**
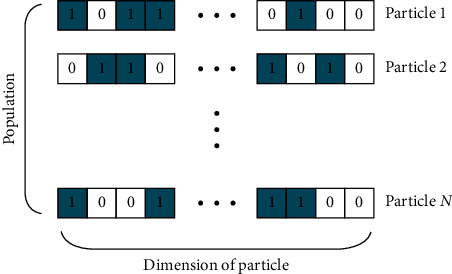
Schematic diagram of the creation of initial population.

Channel and feature selection occurs in discrete search space, so the value of particle in the state space can only be “0” and “1.” The speed update rule of PSO algorithm is still retained, but the position of the particle is determined by the following equation:

(8) $$\begin{array}{c} x_{ij}^{k+1}=\left\{\begin{array}{cc} 1,\; & r<s\left(v_{ij}^{k+1}\right),\\ 0,\; & \text{otherwise,} \end{array}\right. \end{array}$$

where *r* is a random number of [0,  1] and *s*(*v*_*ij*_^*k*+1^) is the sigmoid function. Therefore, the equation indicates that the probability of a particle position value 1 is *s*(*v*_*ij*_^*k*+1^). The PSO optimization process is described as follows:

- Initialize the population. Randomly initialize the position of the population *C*_*N*×*D*_ using binary coding, initialize speed, and set the maximum number of iterations. Here, *N* is the number of particles, and *D* represents the dimension of the particle, which is determined by the number of features to be optimized. Each index represents one feature, where binary “1” represents the feature at the same index that will be used for classification, and “0” indicates that the feature will be ignored.  Figure 3 shows a schematic of the initial population location creation.
- Calculate the fitness of each particle in the population. Fitness value is an indicator used to measure the individual advantages and disadvantages of population. In the experiment, the inverse of the mean square error of the testing data is taken as the fitness function as follows:

(9) $$\begin{array}{c} \text{Fit}\left(x_{ij}^{t}\right)=\frac{1}{\text{mse}\left(\hat{Y}-Y\right)}=\frac{1}{\sum_{i=1}^{n}{\left(\hat{y_{i}}-y_{i}\right)}^{2}}, \end{array}$$

  where $\hat{Y}=\left\{\hat{y_{1}},\hat{y_{2}},\ldots ,\hat{y_{n}}\right\}$ is the predicted value of the testing data, *Y*={*y*_1_, *y*_2_,…, *y*_*n*_} is the true value, and *n* is the number of samples.
(c) Update *p*best and *g*best. For each particle, its fitness value is compared to the fitness value of the best position it has experienced, and *p*best is updated if this value is better. For each particle, its fitness value is compared with the fitness value of the global best position, and if it is better, *g*best is updated.
(d) Update velocity and position. First, the updated velocity *v* is calculated according to equations (5) and (6), where the inertia weight *w* is obtained by (7). Next, the new position is calculated from equation (8).
(e) Repeat steps b–d until the maximum number of iterations is reached, and record the fitness value and *g*best for each iteration to select the best combination of features.

**Figure 4:**
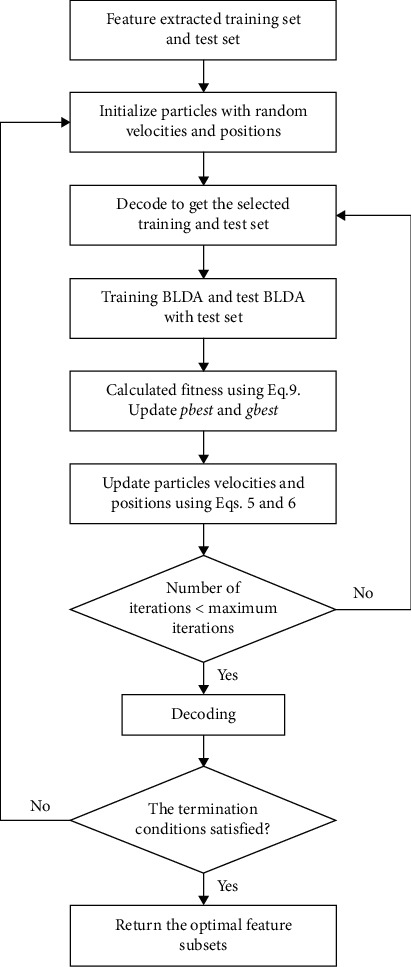
Flowchart of multilevel PSO channel and feature selection.

Figure 4 shows the flow of multilevel PSO for channel and feature selection. The loop is terminated when the maximum execution level is reached or the number of selected features does not change.

### 2.5. Classification Performance

To evaluate the performance of the algorithm, Kappa values, *F*-score, and time-based statistics such as sensitivity (recall), specificity, and precision were used. The Kappa values are an indicator used to measure the accuracy of the classification and can be given by

(10) $$\begin{array}{c} \text{Kappa}=\frac{\text{acc}-\text{rand}}{1-\text{rand}}. \end{array}$$

Here, *acc* is the classification result, and *rand* is the result of random classification. For two-class classification, the value of *rand* is 0.5 [20].

Sensitivity (the proportion of positives that are correctly identified), specificity (the proportion of negatives that are correctly identified), precision (the proportion of correctly predicted positives to all predicted positives), and *F*-score (the harmonic mean of the precision and recall) are defined as follows:

(11) $$\begin{array}{c} \text{sensitivity}=\frac{\text{TP}}{\text{TP}+\text{FN}}\times 100, \end{array}$$

(12) $$\begin{array}{c} \text{specificity}=\frac{\text{TN}}{\text{TN}+\text{FP}}\times 100, \end{array}$$

(13) $$\begin{array}{c} \text{precision}=\frac{\text{TP}}{\text{TP}+\text{FP}}\times 100, \end{array}$$

(14) $$\begin{array}{c} F-\text{score}=\frac{\text{precision}\times \text{recall}\times 2}{\text{precision}+\text{recall}}\times 100, \end{array}$$

where true positive (TP) and true negative (TN) represent the numbers of left-hand little-finger movements and tongue motions, respectively, correctly classified by the algorithm. If these data are erroneously detected as the opposite movements, they are termed as false positive (FP) and false negative (FN).

## 3. Results

### 3.1. Parameter Settings

The scale factors *p* and *q* are used to adjust the width and height of the Gaussian window of the MST by continuously adjusting the value of the scale factor; the results are shown in Table 1, and the most effective features could be obtained when *p*=0.85 and *q*  = 1.

Because the two-level PSO feature selection scheme is very representative, it not only achieves higher classification accuracy but also saves a lot of time compared with multilevel optimization. Therefore, the two-level PSO is taken as an example to illustrate the parameter adjustment process, as shown in Table 2; the optimal parameters are *N* = 100, *c*_1_ = *c*_2_ = 1.5, *w*_max_ = 0.8, *w*_min_ = 0.4, and *v*_max_ = 20. In the experiment, we found that MLPSO usually reached the optimal value within 100 iterations. Therefore, take  *T* = 100. All experiments were started with the initial particle swarms independent of each other.

### 3.2. Channel Selection

**Figure 5:**
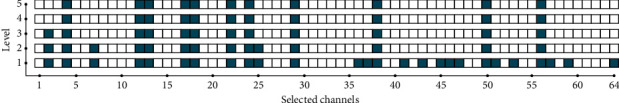
Distribution of selected channels with different levels of PSO for channel selection.

**Figure 6:**
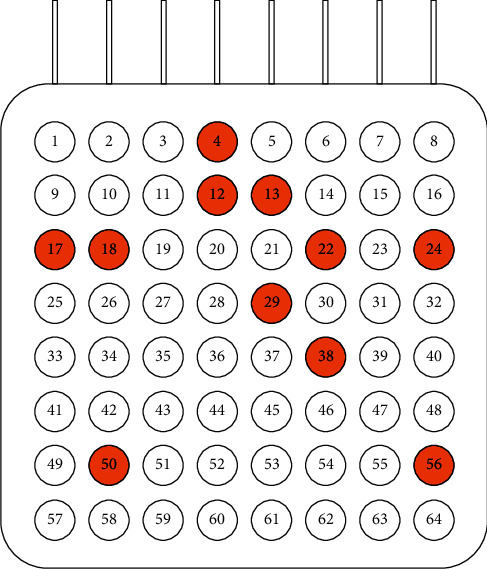
Channel distribution for optimal channel combination.

To confirm the effectiveness and feasibility of the proposed MLPSO optimization framework, we fixed the feature sets representing each channel and utilized the PSO algorithm to find the best channel combination. Results are shown in Table 3. The accuracy of classification increased from 89% to 97%.  Figure 5 shows the distribution of selected channels with different levels of PSO for channel selection. Eleven channels were stable in each experiment. Since all experiments were started with independent initial particle swarms, these 11 channels were considered to be the optimal channel combination, far less than the initial 64 channels.  Figure 6 illustrates the positional arrangements of the corresponding electrodes. The selected channels are relatively concentrated in the upper half, while channels are almost entirely absent from the lower left part.

The electrodes of the dataset used in this study were placed under the dura of the cerebral cortex, covering the main motor area and premotor area, as well as the frontotemporal area of the left and right hemispheres [15]. Therefore, the selected channels were located close to the motor cortex region of the brain. The unselected channels may be due to the interval of a week between collection of the training set and test set, electrode shedding, or decreased conductivity, resulting in poor signal quality.

### 3.3. Feature Selection

**Figure 7:**
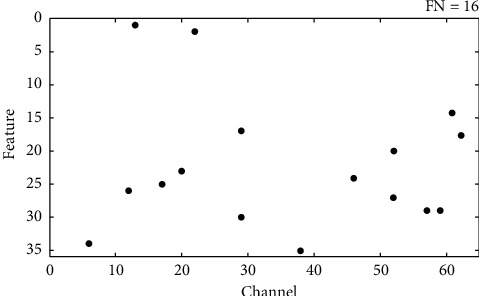
Feature selection scatterplot. The abscissa represents 64 channels in total, the ordinate represents 35 features extracted from each channel, and the black dot represents the selected feature's location.

The results of feature selection are listed in Table 4. After feature selection, the classification accuracy of all experiments was improved by more than 4%, and the best accuracy level reached 99%. In addition, the number of features used for classification was 40 when optimal accuracy was reached for the first time, which was only 1.8% of the original number of features. This coincided with a 95% reduction in testing time. The number of selected features in the last several experiments of the scheme remained unchanged, suggesting that the optimal feature set related to the task was identified.  Figure 7 represents a scatter diagram of optimal feature distribution.

### 3.4. Channel and Feature Selection

Since the optimal channel combination was identified via four-level PSO, we conducted four groups of feature selection experiments using different levels of PSO for channel selection; results are shown in Table 5. The best classification accuracy achieved in each group of experiments was 99%; and the adequate the channel selection, the shorter the feature selection time needed to achieve the greatest accuracy. Concurrently, the specificity of each experiment reached 100%.

## 4. Discussion

### 4.1. Comparison of Channel and Feature Selection

Tables 3–5 show that when only MLPSO is used for channel selection, the best classification accuracy is 97%, while the other two schemes achieved 99% accuracy. This is a significant improvement in accuracy compared to 89% before optimization. At the same time, the number of features used to achieve 99% classification accuracy was less than 10.5% of the original number, and the test time was reduced by more than 90%. These data suggest that MLPSO-based optimization framework not only significantly improves classification accuracy but also effectively reduces the number of features, thus greatly reducing the test time. These characteristics indicate that MLPSO may be useful as a reference for related real-time BCI application system research.

**Figure 8:**
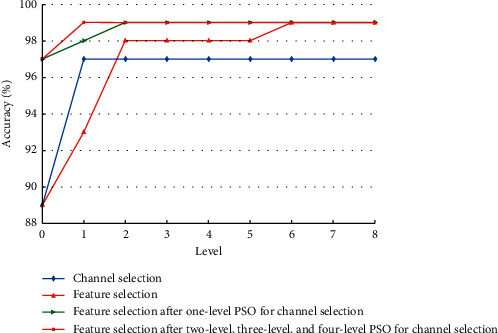
Classification accuracy of each experiment corresponding to different levels of PSO.

Figure 8 shows the change in classification accuracy of each experiment when different levels of PSO are used. Compared with the scheme using feature selection only, use of channel and feature selection requires fewer feature selection times to achieve 99% accuracy. Meanwhile, the total training time of channel and feature selection scheme is less than that of feature selection scheme. This demonstrates that channel selection can filter out channels that are not related to the task and simplify the complexity of the optimal feature selection process. These data suggest that channel and feature selection can accelerate the convergence of the algorithm to the global optimal value, reduce computational complexity, and shorten the training time.

### 4.2. Comparison of the Classification Performance

Tables 3–5 also provide evaluation of classification performance corresponding to each experiment, mainly in terms of Kappa, *F*-score, sensitivity, specificity, and precision. The optimal Kappa value of feature selection scheme based on PSO was 0.98, which demonstrated 20% improvement compared with the method without optimization. Moreover, the accuracy and sensitivity of the PSO-based method were greatly improved, and the specificity reached 100%. These improved evaluation indices indicate the effectiveness of the proposed scheme.

### 4.3. Comparisons with Other Methods

Table 6 presents a comparison of the proposed method with the current state-of-the-art scheme using the same dataset. The classification accuracy of the proposed framework is evidently higher than that of the previously used algorithms. Chang et al. [6] proposed a feature selection scheme based on a genetic algorithm; the classification accuracy of the algorithm is 96%, and the number of selected features is 48.6% relative to the number of original features. By contrast, our scheme achieves 99% accuracy with less than 10.5% features, which proves the effectiveness of the scheme proposed in the present study. Xu et al. [21] proposed using gradient boosting to classify brain signals by extracting the combined features of fractal measures and LBP operators; 41 channels with the highest precision were selected, yielding 95% accuracy. Zhao et al. [22] used band power for channel selection and feature extraction. Eleven channels with distinctive features were selected from the initial channel. Principal component analysis was used to reduce the dimensions of features. Finally, FLDA was used for classification, achieving 94% accuracy, but the algorithm has high complexity. Ince et al. [23] proposed an adaptive classification scheme, including the generation of a structured redundant feature dictionary based on dual-tree undecimated wavelet packet transform (UDWT) and linear discriminant analysis (LDA) classifiers. By using only three features, 93% accuracy can be achieved, but the subset of its features will increase algorithm complexity. Wei et al. [24] selected the optimal channel through the genetic algorithm, and then the common spatial pattern (CSP) extracted the power characteristics, and FLDA classified it to achieve 90% classification accuracy through seven channels. Compared with other methods, our algorithm has high classification accuracy and specificity.

## 5. Conclusions

This study describes three optimization schemes for motor imagery-based BCI. MLPSO is used to optimize the process of channel and feature selection, channel selection, and feature selection, respectively, and MST-based PSD and BLDA were used for feature extraction and classification. The scheme of using MLPSO for feature selection and hybrid channel-feature selection achieved 99% classification accuracy, the test time was shortened by more than 90%, and Kappa values were increased from 0.78 to 0.98, and the specificity reached 100%, achieving the best reported level. The results show that the channel and feature selection scheme can accelerate the speed of finding the global optimal value and reduce the training time. Due to the excellent performance of the proposed optimization scheme, it can provide a reference for related real-time BCI application system research.

## Figures and Tables

**Table 1:** Classification results under different scale factor values of MST.

*p*	*q*	Accuracy (%)^a^
0.7	1	86
0.8	1	88
0.85	1	89
0.9	1	88
1	1	87
0.85	0.8	84
0.85	0.9	87
0.85	1.1	86
0.85	1.2	82

^a^PSO is not used for optimization during classification.

**Table 2:** Parameter setting and classification result of two-level PSO for feature selection.

	PSO parameters							Results		Runtimes	
No.	*N*	*T*	*c*_1_	*c*_2_	*w*_max_	*w*_min_	*v*_max_	NF	ACC (%)	Training time (s)	Test time (s)
1	100	100	1.5	1.5	0.8	0.4	20	541	98	13,717	1.089
2	100	100	1.3	1.3	0.8	0.4	20	556	94	13,637	1.125
3	100	100	1.7	1.7	0.8	0.4	20	542	94	13,720	1.154
4	100	100	1.5	1.5	1.0	0.4	20	555	94	13,753	1.108
5	100	100	1.5	1.5	1.0	0.2	20	580	94	13,701	1.176
6	100	100	1.5	1.5	0.8	0.2	20	544	95	13,938	1.146
7	100	100	1.5	1.5	0.8	0.4	18	572	95	13,700	1.125
8	100	100	1.5	1.5	0.8	0.4	22	564	94	13,579	1.128
9	80	100	1.5	1.5	0.8	0.4	20	580	96	10,812	1.139
10	120	100	1.5	1.5	0.8	0.4	20	587	96	15,862	1.167

No.: number; N: population size; T: maximum number of iterations; NF: number of selected features; ACC: accuracy.

**Table 3:** Classification results with different levels of PSO for channel selection.

Level	SC	ACC (%)	TRT (s)	TET (s)	Kappa	*F*-score	SEN	SPE	PRE
1	26	95	7,258	1.215	0.90	95.14	98	92	92.45
2	14	97	9,862	0.986	0.94	97.03	98	96	96.08
3	12	97	11,109	0.854	0.94	97.03	98	96	96.08
4	11	97	13,270	0.791	0.94	97.03	98	96	96.08
5	11	97	15,248	0.791	0.94	97.03	98	96	96.08

SC: selected channels; ACC: accuracy; TRT: training time; TET: test time; SEN: sensitivity; SPE: specificity; PRE: precision.

**Table 4:** Classification results with different levels of PSO for feature selection.

Level	SF	ACC (%)	TRT (s)	TET (s)	Kappa	*F*-score	SEN	SPE	PRE
0^a^	2,240	89	46	2.268	0.78	89.72	84.21	95.35	96
1	1,098	93	8,743	1.455	0.86	93.33	89.09	97.78	98
2	541	98	13,717	1.089	0.96	98	98	98	98
3	276	98	13,835	0.255	0.96	98	98	98	98
4	133	98	14,227	0.163	0.96	98	98	98	98
5	69	98	15,263	0.122	0.96	98	98	98	98
6	40	99	16,373	0.104	0.98	98.99	98	100	100
7	23	99	17,840	0.105	0.98	98.99	98	100	100
8	20	99	17,917	0.099	0.98	98.99	98	100	100
9	16	99	18,075	0.096	0.98	98.99	98	100	100
10	16	99	18,116	0.096	0.98	98.99	98	100	100

^a^Without PSO. SF: selected features; ACC: accuracy; TRT: training time; TET: test time; SEN: sensitivity; SPE: specificity; PRE: precision.

**Table 5:** Classification results with different levels of PSO for feature selection after channel selection.

Level	SF	ACC (%)	TRT (s)	TTT (s)	TET (s)	Kappa	*F-*score	SEN	SPE	PRE
*Feature selection after one-level PSO for channel selection*										
1	434	97	2,520	9,778	0.892	0.94	97.03	98	96	96.08
2	215	98	4,024	11,300	0.198	0.96	98	98	98	98
3	125	99	4,629	11,887	0.153	0.98	98.99	98	100	100
4	54	99	4,887	12,145	0.107	0.98	98.99	98	100	100
5	37	99	5,058	12,316	0.103	0.98	98.99	98	100	100
6	28	99	5,299	12,557	0.101	0.98	98.99	98	100	100
7	17	99	5,417	12,675	0.098	0.98	98.99	98	100	100
8	17	99	5,530	12,788	0.098	0.98	98.99	98	100	100

*Feature selection after two-level PSO for channel selection*										
1	263	98	1,443	11,305	0.241	0.96	98	98	98	98
2	122	99	2,106	11,968	0.149	0.98	98.99	98	100	100
3	56	99	2,389	12,251	0.112	0.98	98.99	98	100	100
4	33	99	2,572	12,434	0.102	0.98	98.99	98	100	100
5	25	99	2,703	12,565	0.101	0.98	98.99	98	100	100
6	20	99	2,818	12,680	0.099	0.98	98.99	98	100	100
7	12	99	2,913	12,775	0.095	0.98	98.99	98	100	100
8	12	99	3,005	12,867	0.095	0.98	98.99	98	100	100
*Feature selection after three-level PSO for channel selection*										
1	235	99	1,437	12,546	0.225	0.98	98.99	98	100	100
2	133	99	2,225	13,334	0.163	0.98	98.99	98	100	100
3	66	99	2,586	13,695	0.131	0.98	98.99	98	100	100
4	36	99	2,805	13,914	0.107	0.98	98.99	98	100	100
5	26	99	2,984	14,093	0.103	0.98	98.99	98	100	100
6	16	99	3,136	14,245	0.096	0.98	98.99	98	100	100
7	15	99	3,275	14,384	0.096	0.98	98.99	98	100	100
8	15	99	3,391	14,500	0.096	0.98	98.99	98	100	100

*Feature selection after four-level PSO for channel selection*										
1	196	99	958	14,228	0.189	0.98	98.99	98	100	100
2	107	99	1,451	14,721	0.151	0.98	98.99	98	100	100
3	58	99	1,710	14,980	0.122	0.98	98.99	98	100	100
4	28	99	1,861	15,131	0.101	0.98	98.99	98	100	100
5	18	99	1,962	15,232	0.099	0.98	98.99	98	100	100
6	18	99	2,068	15,338	0.099	0.98	98.99	98	100	100
7	17	99	2,150	15,420	0.097	0.98	98.99	98	100	100
8	17	99	2,236	15,506	0.097	0.98	98.99	98	100	100

SF: selected features; ACC: accuracy; TRT: training time; TTT: total training time of channel selection and feature selection; TET: test time; SEN: sensitivity; SPE: specificity; PRE: precision.

**Table 6:** Comparison between our proposed method and existing methods.

Feature extraction	Channel selection	Feature selection	Classification	Accuracy (%)
MST	√	√	BLDA	99
ST [6]	—	√	BLDA	96
*LBP*_4,1_^*riu*2^, *LBP*_8,1_^*riu*2^, fractal intercept, and lacunarity [21]	√	—	Gradient boosting	95
Band power [22]	√	—	FLDA	94
UDWT [23]	√	√	LDA	93
CSP [24]	√	—	FLDA	90

## Data Availability

The BCI Competition III dataset I is available at http://bbci.de/competition/iii/.
